# Stochastic model of lignocellulosic material saccharification

**DOI:** 10.1371/journal.pcbi.1009262

**Published:** 2021-09-13

**Authors:** Eric Behle, Adélaïde Raguin

**Affiliations:** Department of Biology, Cluster of Excellence on Plant Sciences, Institute of Quantitative and Theoretical Biology, Heinrich-Heine University, Düsseldorf, Germany; University of Illinois at Urbana-Champaign, UNITED STATES

## Abstract

The processing of agricultural wastes towards extraction of renewable resources is recently being considered as a promising alternative to conventional biofuel production. The degradation of agricultural residues is a complex chemical process that is currently time intensive and costly. Various pre-treatment methods are being investigated to determine the subsequent modification of the material and the main obstacles in increasing the enzymatic saccharification. In this study, we present a computational model that complements the experimental approaches. We decipher how the three-dimensional structure of the substrate impacts the saccharification dynamics. We model a cell wall microfibril composed of cellulose and surrounded by hemicellulose and lignin, with various relative abundances and arrangements. This substrate is subjected to digestion by different cocktails of well characterized enzymes. The saccharification dynamics is simulated *in silico* using a stochastic procedure based on a Gillespie algorithm. As we additionally implement a fitting procedure that optimizes the parameters of the simulation runs, we are able to reproduce experimental saccharification time courses for corn stover. Our model highlights the synergistic action of enzymes, and confirms the linear decrease of sugar conversion when either lignin content or crystallinity of the substrate increases. Importantly, we show that considering the crystallinity of cellulose in addition to the substrate composition is essential to interpret experimental saccharification data. Finally, our findings support the hypothesis of xylan being partially crystalline.

## Author summary

Leftover wastes generated by agriculture, such as inedible leaves and stalks of plants, represent an abundant and unexploited raw material that contains energy in the form of sugar polymers. Their breakdown and processing into bio-ethanol is recently being considered as a promising candidate for renewable fuel production. However, it is still poorly understood, how the microscopic structure and composition of plant waste materials impact their enzymatic digestion. Various experimental pre-processing methods are currently being tested to determine their effect on the material composition and structure, and the sugar conversion. In this study, we present a computational model to complement such experimental approaches. We simulate a microscopic plant fragment typically found in plant waste materials, whose structure and composition can be tailored. This fragment is then subjected to enzymatic digestion, whose dynamics is tracked *in silico*. The model reproduces experimentally observed time courses for plant fragments of known composition. It additionally provides new hypotheses for interpreting complex experimental results.

## 1 Introduction

The worldwide challenges of energy supply and resource shortage are becoming ever more urgent. Utilizing renewable resources such as waste biomass generated via the agricultural industry is an inviting alternative to face these difficulties [1]. Lignocellulosic materials which are currently considered waste, for example those parts of crops which are not applicable for animal or human consumption, are of particular interest. They contain large amounts of chemical energy, which may be utilized in processing sectors such as the biofuel industry. Strategies for energy extraction include high-temperature conversion to syngas [2], fast pyrolysis [3], and bioconversion methods [4, 5], each of which is still an area of active research. We focus here on the bioconversion methods. The objective is to extract monomeric sugars from the many polysaccharides found within lignocellulosic material, and then to ferment them into ethanol. The process of extracting these sugars is called saccharification [6], and in our case is carried out via enzymatic digestion [7].

Efficient enzymes for sugar extraction can be found in microorganisms which are specialized towards plant digestion [8]. Utilizing them for human benefit has been studied extensively, and these enzymes are well characterized [8, 9]. However, this strategy is so far costly, as it requires isolation of large quantities of enzymes [1]. As a result, various pre-treatment methods have been conceived [10–12]. Their purpose is to alter the substrate structure in order to improve ease of access for the enzymes to the valuable sugars. The methods used include pre-treatment with acids such as H_2_SO_4_ [12] or HCl [10], as well as exposure to steam at various temperatures and exposure times [11]. The impact of pre-treatment severities and substrate structural properties on saccharification dynamics is central in our study. Through the presentation of the model construction in section 2, we explain the biological ground of our assumptions and detail the experimental findings that motivate them.

To complement experiments, modeling approaches have been used to simulate the structure of lignocellulosic materials at different length scales. Due to the early interest in wood, for instance as a construction material, models of lignocellulose structure have been investigated for decades. They can be classified depending on their scale of study, and have been reported in the recent and comprehensive review by Ciesielski et al. [13]. Studies at the lowest scale have triggered much interest, they allow to tackle diverse questions that span from pyrolysis [14, 15], to enzyme mechanisms [16, 17]. They focus on atomistic and molecular methods. These are all-atom models based on density functional theory and quantum/molecular mechanics. Unfortunately, despite remarkable improvements in computational power and high performance computer facilities, molecular dynamics methods are hardly able to depict biopolymers at the nanoscale. An illustration of the typical upper limit has been provided by Vermaas et al. in 2015 [18]. The authors simulated a box of 95 nm x 62,5 nm x 62,5 nm containing an array of 9 lignocellulose microfibrils (length of 160 monomers each) surrounded by 54 cellulases, lignin, water and sodium ions. Together this constituted up to 23.7 milion atoms and could be simulated for a real-time duration of 1.4 microseconds. Besides, Charlier and Mazeau [19] proposed a molecular dynamics model including cellulose, xylan, water, and lignin. They showed that xylan and cellulose display a well-defined interface, while some xylan could interpenetrate into the lignin part. However, this model did not consider saccharification. Some coarse-grained molecular dynamics methods have also been pursued by using beads or pseudo-atoms as elementary units. They aim at overcoming the limitations of all-atom models, as explained by Ingólfsson et al. [20]. So far this method remains limited, and does not address interactions between polymers. At a larger scale, from about 10^−5^ to 10^−1^ m, many models for biomass conversion focus on reaction-diffusion at varying temperatures. For instance, they investigate the effect of particle’s pore size and structure on the diffusion of acid during pre-treatments and enzymes during saccharification [21–23]. Models that consider much larger scale, such as whole-reactor, are mostly dedicated to high-temperature conversion processes like pyrolysis and gasification. These are reviewed by Basu and Kaushal [24], but are not related to enzymatic saccharification. In parallel, since the 1970’s a multitude of kinetic models have been proposed for the saccharification of cellulose, including models derived from Michaelis-Menten kinetics, Langmuir adsorption kinetics on a surface, and fractal kinetics. The latter tends to better reflect the spatial constraints of the media. Kinetics that reflect the synergism of cellulases have also been greatly investigated. Bansal and coworkers published a well structured review that can rapidly introduce a curious reader to these methods [25].

Despite the diversity of the approaches and the richness of the field, so far the substrate’s properties in terms of structure, composition, size and crystallinity are mainly under-investigated in modeling, even though those are identified as major factors for saccharification [13]. Two central challenges are, on the one hand, to consider the variability and complexity of the plant material substrate, and on the other hand, to capture the space and time scales of the saccharification process. A few studies attempt to tackle these challenges by developing meso-scale models of saccharification for coarse-grained substrates and intermediate time scales. In 2013, Kumar and Murthy [26] combined experimental and theoretical methods to investigate the effect of enzyme crowding. They built a stochastic Monte Carlo algorithm and simulated the action of endoglucanase, cellobiohydrolase, and *β*-glucosidase on a bundle of cellulose microfibrils. Although they took into consideration crystallinity for the outermost regions of the substrate, they did not vary it in order to characterize its impact on the saccharification dynamics. In 2017, they published a refined version of their model [27] that showed improved comparison to experimental results. Still, in both studies the discrepancy between experimental and simulation results was noticeable, and the substrate modelled contained only cellulose. Lignin and hemicellulose were not part of it. Vetharaniam et al. [28] developed a 3-D, agent-based model of perennial ryegrass mesophyll cell wall digestion by Cel51A, Cel9D and endoxylanase 1 enzymes. The model accounted for cellulose crystallinity and hemicellulose sugars. The synergy of the different enzymes was extensively discussed but simulated results were not quantitatively compared to experimental data. Crystallinity was not varied in order to discuss its impact on the saccharificaiton dynamics, and since the model focused on primary cell wall, lignin was excluded. Finally, Asztalos et al. [29] studied the synergistic action of multiple enzymes on cellulose. They set a detailed agent-based model that includes several steps such as adsorption of cellulases on the solid cellulose substrate, inter-chain hydrogen bond breaking, hydrolysis of glycosidic bonds, and desorption of cellulases from cellulose. Their model is however limited to surface reactions and the substrate is simplified to a two dimensional grid of glucose. In this study, hemicellulose and lignin are also not investigated. It is therefore clear that a comprehensive model which simulates lignocellulose enzymatic saccharification by focusing on the substrate properties in close comparison with experimental data is so far missing.

In this study we present a computational model of lignocellulose digestion whose purpose is precisely to suppress simultaneously and interdependently address the so-far under-investigated questions of structure, composition and crystallinity of the substrate. Although certain preceding models capture some of those aspects, to the best of our knowledge, none of them embrace comparable scope of parameters, range of questions and ability to reproduce experimental data. To develop such a model we need to represent the biochemistry and the biophysics of the system at length scales of hundreds of nm and time scales of hours, and at the same time cope with finite computational power. We therefore build a coarse-grained model based on stochastic simulations alike Kumar and Murthy [26, 27] and Asztalos et al. [29]. Throughout, we follow a purely theoretical approach supplemented by abundant experimental data found in the literature. A major aim is to understand the effect of the substrate structure on the action of the enzymes as well as their interaction with non-digestible lignocellulose components. The model accounts both for the composition and three-dimensional structure of the substrate, and the distinct enzymes typically used as cocktails in industry.

The manuscript is organized as follows: in section 2 we present the model in detail in terms of the underpinning biological system and its computational implementation. This includes to briefly review the principles of the stochastic Gillespie algorithm. In section 3, we explain in detail the simulation conditions, and in particular motivate the enzyme concentration implemented. In section 4, we present the model’s results in light of experimental measurements. We decipher the enzyme activity and characterize the influence of the lignin content and the crystallinity on the saccharification process. Then, we utilize published experimental data by Bura et al. [11] to rationalize saccharification time courses for corn stover samples pre-treated to different extent. This enables us to infer the critical role of substrate structure and, in particular, substrate crystallinity. In section 5, we discuss our results and consider further potential developments of our modeling approach.

Extensive technical details on our computational approach are provided on our public GitLab repository (“https://gitlab.com/erbeh/pcwsm”). This includes details on the Gillespie algorithm, clean and commented codes, instructions on how to run them, explanations of the content of the input and output files of the simulation code, and details on our optimization procedure to fit experimental saccharification curves. Also, we provide all *in silico* data and scripts necessary to produce the figures presented here.

## 2 The model

We built a complex computational model that constitutes a general platform to investigate different plant mutants, tissues and enzyme abundances and kinetics. In this section, we provide details on the biological system and features included in the model in terms of substrate and enzymes. We also clarify the assumptions we make towards reducing the computational cost of the model. We first discuss plant cell wall composition and structure, then enzyme cocktails, substrate-induced effects, and finally we briefly introduce the Gillespie algorithm.

### 2.1 Plant cell wall composition and structure

The plant cell wall is a complex structure usually consisting of a primary and secondary layer [30]. Each layer contains different amounts of proteins and structural polymers. The microstructure, i.e., the composition and arrangement of the components, varies strongly between different cell types, and both the individual cell composition and the multicellular arrangement strongly influence the mechanical properties of the plant [30]. In this study we focus on the three types of biopolymers found primarily within plant cell walls: cellulose, hemicellulose and lignin. In line with the field of applications connected to our study (biofuel industry), we consider an averaged plant cell wall composition, in which the primary and secondary cell walls are not distinguished.

Cellulose is the most abundant organic polymer found on earth [30, 31]. It functions as a structural backbone within the cell wall and is composed of linear chains of up to 15 000 glucose molecules connected via β(1 → 4) glycosidic bonds [30, 32, 33]. Cellulose polymers adopt a linear shape even at high degree of polymerization, and therefore represent a favorable structural backbone due to their rigidity. For further stabilization of the linear chains, bundles of multiple cellulose polymers are formed within the cell wall, so-called microfibrils [30, 34, 35]. These are distributed throughout the cell wall with varying degrees of order in their alignment [34].

**Fig 1:**
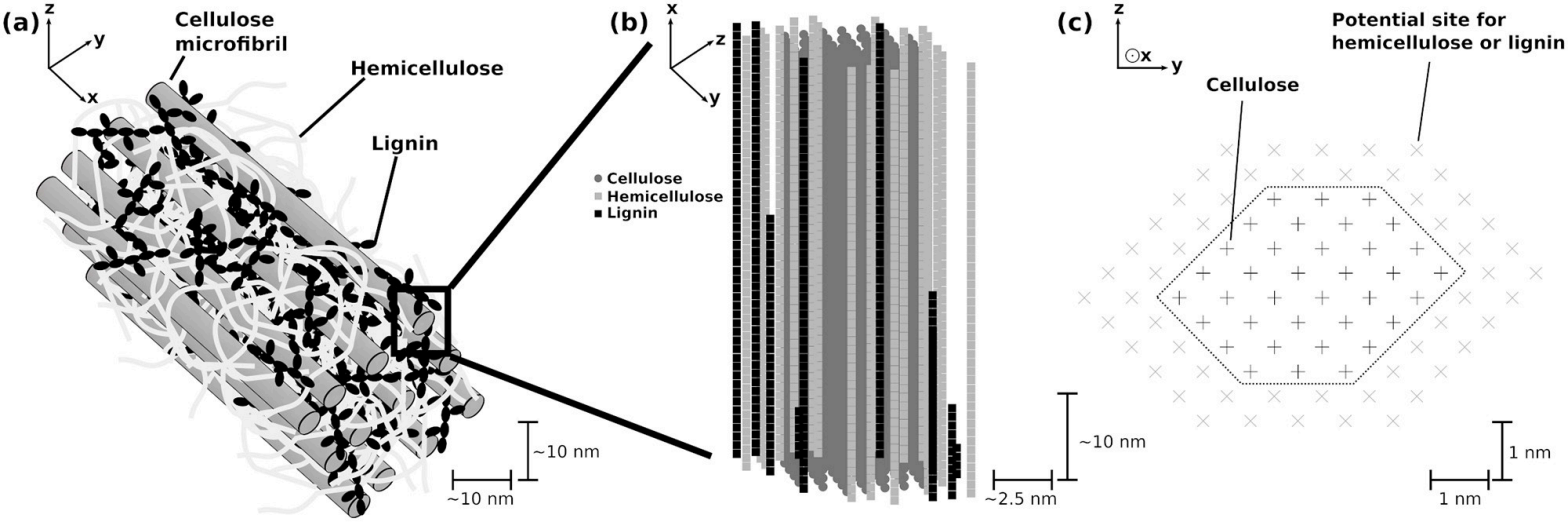
Schematic representation of the structure of the substrate in the model. (a) Several cellulose microfibrils embedded in a matrix of hemicellulose reinforced by lignin. (b) Side-view of the top 50 nm of a single cellulose microfibril made of 36 polymers, and part of the surrounding matrix showing the relative arrangement of the hemicellulose and lignin polymers as well as gaps. In (a) and (b), polymer types are color-coded (cellulose: dark gray; hemicellulose: light gray; lignin: black). (c) Top-down view of the structure shown in (b). The core enclosed by the dotted line is composed of 36 polymers of cellulose (black crosses), and its structure follows that used by Ding et al. [57]. The positions in the two outer layers (gray crosses) can each either be hemicellulose or lignin polymers.

Hemicellulose polymers consist of short chains of multiple types of sugar monomers [36, 37], whose total number per polymer lies between 50 and 200 [38–41]. The latter can be substituted by acetyl esters. The function of hemicellulose polymers within the cell wall matrix is the anchoring of the cellulose microfibrils embedded within it (see Fig 1A). The definition of hemicellulose is not fully consistent across literature. Therefore, we use that of Pauly et al. [37], where any non-cellulose cell wall polysaccharide whose dominant backbone is linked by β(1 → 4) glycosidic bonds is considered hemicellulose. Several distinct polysaccharides fit this definition. As a result, hemicellulose is further divided into four basic types [36, 37]: xylans, mannans, mixed linkage β-glucans, and xyloglucans. Each of them is characterized by different sugar composition, as well as differences in their three-dimensional structure. Here we focus on a single type of hemicellulose: the xylans. Xylans are the most abundant hemicellulose polymers [42–44]. They are characterized by a backbone composed of the five-carbon monosaccharide xylose [37], which may be partially branched by glucuronic acid or L-arabinose, via α(1 → 6) glycosidic bonds [37]. Xylans mainly occur within the secondary cell wall and act as further structure reinforcement [37].

Lignin is the second most abundant biopolymer after cellulose [45–47]. It is a manifold branched polymer [48–50], which is known for being a strong contributor to the mechanical properties of wood. The lignin structure is mainly composed of three monomers (monolignols) [45]. The most common monolignols differ according to methoxy groups attached to the aromatic ring.

In the model, we retain the main features of the cellulose, hemicellulose and lignin polymers. The simulated substrate is composed of a cellulose microfibril around which hemicellulose and lignin polymers are randomly arranged and form up to four outer layers (see Fig 1C, where two of the four layers are shown). The outer layers are not fully covering, but possess gaps (see Fig 1B), which represent the non-complete surrounding of the cellulose by hemicellulose and lignin. The length of the microfibril is specified in terms of the number of glycosidic bonds within an individual cellulose polymer at the start of the simulation. While plant cell wall cellulose can have a degree of polymerization (DP) in the thousands [51, 52], because of computational power limitations we investigated a DP of 200. Although it is still much shorter than the natural length of a cellulose polymer in plants, our assumption is supported by the fact that we are interested in industrial processes that include pre-treatments, and that pre-treatments strongly reduce the DP of cellulose microfibrils [52]. Since the shape of the microfibril, as well as the number of cellulose polymers within it, may vary for different types of substrate (e.g. 18 or 24 polymers in mung beans [53], spruce wood [54] and celery collenchyma [34]; 36 polymers in maize plants [55–57]), these parameters can also be freely specified in our model. In this study, we aim to simulate material from maize plants, and therefore choose a microfibril composed of 36 cellulose polymers, arranged in a quasi-hexagonal shape [55–57] (see Fig 1C).

**Fig 2:**
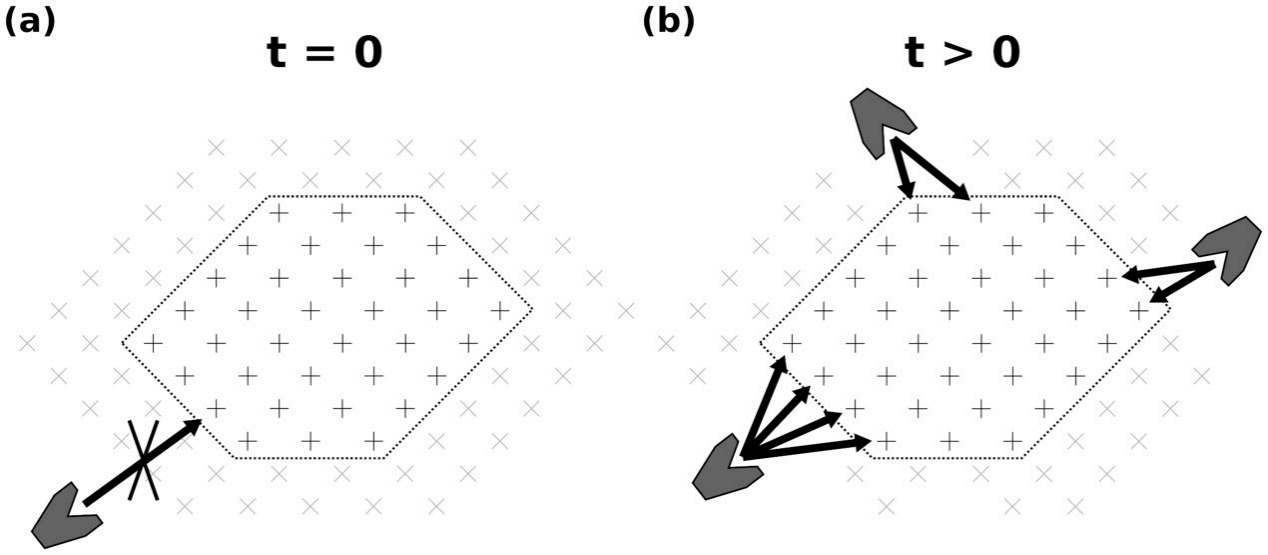
Sketch (top-down view) of the accessibility of polymer bonds depending on their position within the substrate and its digestion state. (a) Beginning of the simulation: the outer layer (gray crosses, here made entirely of hemicellulose) does not contain any gaps. None of the cellulose bonds (black crosses) are accessible for digestion by enzymes (dark gray polygons). Only the hemicellulose bonds are digestible. (b) Later stage of the simulation: some of the hemicellulose within the outer shell has been digested. The cellulose bonds highlighted by arrows are now accessible for digestion.

The structure of the substrate is resolved in three dimensions at the scale of monomers: glucose for cellulose and xylose for hemicellulose. For lignin, monomers are a representative monolignol, which is sufficient to retain the effects of lignin we investigate (enzyme adhesion and structure blocking, see section 2.3). We simulate cellulose and hemicellulose as linear polymers, but we mimic the branched structure of lignin. The composition of the substrate in terms of polymer percentages may be tuned, and the percentages of cellulose, hemicellulose and lignin sum up to 1. In the initial system state, the cellulose polymers have the same length, but due to the gaps within the surrounding layers the hemicellulose and lignin polymers do not. All bonds within the cellulose and hemicellulose polymers are potentially subject to enzymatic digestion, unlike lignin that cannot be digested by the enzymes considered here. A bond can only be digested if it is located at an accessible position, which is the case as soon as it is exposed to the medium surrounding the microfibril (see Fig 2).

### 2.2 Enzyme cocktails

Enzymes are a central focus for the optimization of plant cell wall saccharification [58, 59]. Several digesting enzymes have been isolated from nature and characterized, such that their mode of action on simple linear and soluble polysaccharides is well known [60–63]. For instance, species belonging to the *Trichoderma* genus excrete two groups of enzymes, respectively called cellulases and xylanases [8]. These sets of enzymes are typically used by industry to process raw plant material [64] and thus produce glucose from lignocellulosic biomass. We choose to focus on those in the model.

**Fig 3:**
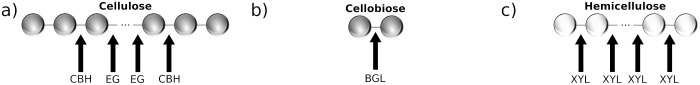
Schematic representation of the polymer bonds that can be digested within the model. (a) Cellulose digestion sites by endoglucanase (EG) and cellobiohydrolase (CBH). Endoglucanase may digest glucose-glucose bonds along the entire polymer, except for the two outermost bonds at each end. Cellobiohydrolase processively cuts off cellobiose from either polymer ends. (b) Cellobiose digestion site by β-glucosidase (BGL). β-glucosidase exclusively digests cellobiose into two glucose molecules. (c) Hemicellulose digestion sites by xylanase (XYL). Xylanase may digest xylose-xylose bonds at any position along the hemicellulose polymer.

Endoglucanases (EG) are capable of binding to any position along a cellulose polymer and digesting the β(1 → 4) glycosidic bond between two neighboring glucose molecules [8, 65]. They thereby cut the polymer into two shorter ones. The behavior of endoglucanases with respect to short polymers is not well understood. However, Scapin et al. [63] have described a bacterial endoglucanase which only digests cellulose polymers of a length of at least five glucose units. In the model, due to the uncertainty of the behavior of EG on short polymers we assume that EG mainly acts within the bulk of cellulose polymers, where it can digest any exposed bond. Therefore, it cannot digest the two outermost bonds on each polymer end (see Fig 3A). This implies that only the action of cellobiohydrolases can lead to the release of cellobiose.

Cellobiohydrolases (CBH) specifically digest the glycosidic bonds at either the reducing or non-reducing ends of cellulose polymers, and cut off cellobiose [8, 65] (see Fig 3A). Contrarily to other enzymes considered in the model, CBH is processive [62]. Brady et al. [62] have measured the average step number *N*_steps,CBH_ and association time *t*_CBH_ of individual *Trichoderma reesei* CBH enzymes [62]. They found the following values:

$$\begin{array}{c} N_{\text{steps},\text{CBH}}\approx 50\;\text{and}\;t_{\text{CBH}}\approx 90\;s. \end{array}$$ (1)

While it is clear that there are multiple variants of cellobiohydrolases with different kinetic values found in nature, we base our simulations on these data for CBH. Using *N*_steps,CBH_ and *t*_CBH_, we can estimate a value for the digestion rate *k*_CBH_:

$$\begin{array}{c} k_{\text{CBH}}=\frac{N_{\text{steps},\text{CBH}}}{t_{\text{CBH}}}\approx 0.56\;s^{-1}\approx 2000\;h^{-1}. \end{array}$$ (2)

Within the model, the CBH enzymes may attach to any exposed polymer end at a rate *k*_CBH,attach_. While moving along the polymer, they release cellobiose at a rate *k*_CBH_. Each enzyme remains attached for a time randomly chosen from a normal distribution centered around *t*_CBH_, unless it reaches the end of the polymer and detaches. Importantly, since CBH enzymes are processive they remain attached to the substrate for longer than diffusive enzymes, and the consequent steric hindrance should be considered in the model. To estimate the enzyme size we use the results by Vermaas et al [18]. They modeled TrCel7A enzymes using a highly resolved molecular dynamics approach, from which we can estimate the enzyme size. In order to do so, we approximate the enzyme shape as hard spheres and we deduce a radius of *R*_CBH_ = 4.25 nm. Thus, as long as a CBH enzyme is attached to the microfibril, no other digestion reactions can take place at a distance closer than 2 ⋅ *R*_CBH_ to its attachment point.

β-glucosidases (BGL) complete the saccharification process by digesting cellobiose into two glucose molecules [65, 66] (see Fig 3B). In the model, BGL’s mechanism is therefore the simplest, and its action is only to digest any exposed cellobiose molecule into two glucose molecules.

Xylan-type hemicellulose is digested by xylanases (XYL), a group of enzymes which act analogously to the cellulases on cellulose, and whose action leads to the release of single xylose molecules [42]. For simplicity, and since our main focus is the digestion of cellulose into glucose, in the model we implement the action of xylanases (XYL) in a coarse-grained fashion, i.e., a single enzyme that represents a cocktail of the xylanase sub-types. The XYL mechanism does not distinguish between hemicellulose polymer lengths or the location of bonds along an individual polymer. It simply digests any exposed bond (see Fig 3C).

In the model, EG, CBH, BGL and XYL are strictly distinct with regard to their mechanism, kinetics and abundance (see Fig 3). While the specific mechanism of each enzyme type is fixed, their kinetics and abundance can be tuned. All enzymes except CBH are implemented as diffusive and are assumed to be homogeneously distributed within the medium over the course of the entire simulation.

### 2.3 Substrate induced effects

Many substrate induced effects are known to influence the efficiency of the saccharification process. The most important ones are considered in our model and discussed below.

#### Lignin adhesion

**Fig 4:**
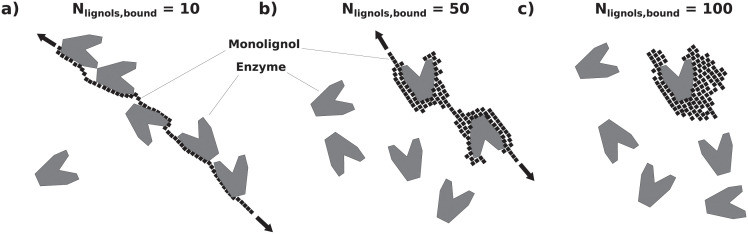
Sketch of the impact of *N*_lignols,bound_ (the number of monolignols involved in binding per bound enzyme) on the total number of enzymes that can be bound. Starting at low values of *N*_lignols,bound_ in (a), many enzymes can be bound to a single lignin polymer. As *N*_lignols,bound_ increases in (b) and (c), this number steeply drops, finally reaching the limit of only a single enzyme being bindable.

Lignin acts as an adhesive towards both cellulases and xylanases, and is thought to negatively affect their action [18, 49, 67, 68]. Previous computational investigations of lignin adhesive properties include the work by Vermaas et al. [18]. Using molecular dynamics simulations, they mimicked an atomic-detail cellulose microfibril surrounded by freely floating cellulases and lignin oligomers. They showed that the lignin oligomers in their system have a high affinity for binding both to the cellulose-binding domain of the investigated cellulase, and to those positions on the cellulose microfibril which are also preferred locations of action by the enzymes. The two effects exhibited by lignin within our model are in line with their conclusions. We consider the adhesion effect towards enzymes, and we additionally include the structural blocking effect resulting from the arrangement of the lignin around the microfibril (see next paragraph). Furthermore, our model allows to investigate how these effects impact the saccharification curves arising from different lignin contents. In the model, we represent the adhesive effect of lignin as an additional event that consists in binding randomly selected enzymes. The binding capacity is finite since the amount of lignin itself is finite. Although all monolignols have the ability to contribute to the binding process, the binding of a single enzyme involves several of them. The number of monolignols which are involved per bound enzyme is a tunable quantity, noted *N*_lignols,bound_. Fig 4 illustrates the cases of 10 monolignols being involved in the binding of each enzyme, as well as 50 and 100 monolignols. The initial amount of lignin monolignols available for binding is the overall number of monolignols *N*_lignols_. Each time that an enzyme is bound to lignin, the number of monolignols available for binding decreases by a value normally distributed around *N*_lignols,bound_, until no more binding can take place. In the model, if the amount of lignin in the system is in large excess compared to the enzymes, they can potentially all be bound, in which case the saccharification process is stopped. Also, the probability for an enzyme to bind to lignin depends on its relative concentration. This means that an enzyme with a lower concentration will be selected for adhesion to lignin less often, and *vice versa*.

#### Structural blocking

**Fig 5:**
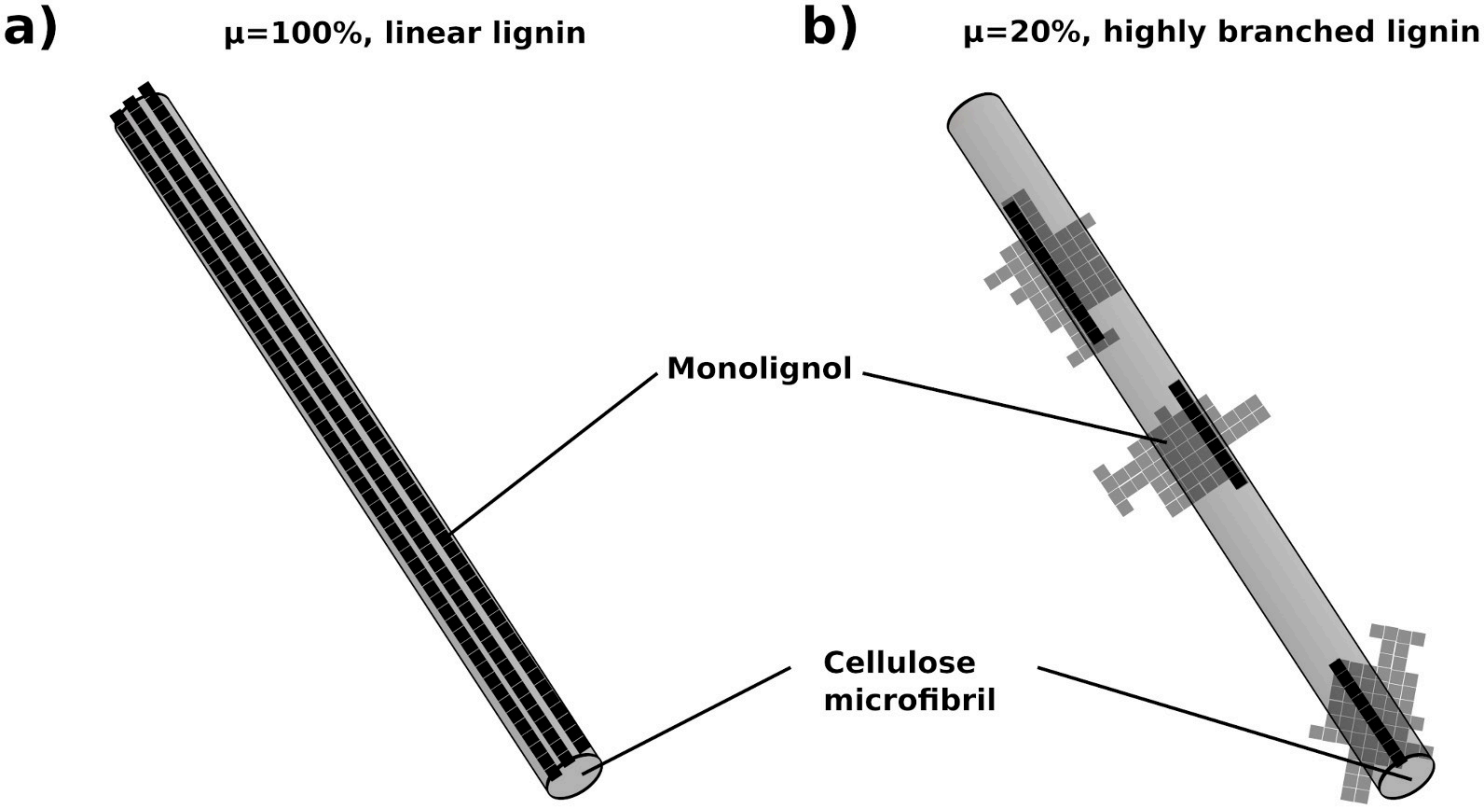
Sketch of the structural blocking by lignin for two different values of the covering fraction *μ*. (a) For *μ* = 100%, every bond within each lignin polymer represents a barrier. The polymers are linear. (b) For *μ* = 20%, only parts of each polymer represents a barrier (showed in black) while remaining monolignols are showed as shades. The polymers are highly branched.

Hemicellulose and lignin partly block the access of cellulases to the cellulose simply by their presence as a physical barrier around the microfibril [11, 18, 69]. Therefore, the arrangement of hemicellulose and lignin has an impact on the saccharification by the cellulases. This is reinforced for lignin, since contrarily to hemicellulose it cannot be digested by the enzymes we consider. In the model, we assume that a cellulose bond covered by lignin cannot be digested until an adjacent bond has been digested, providing access to it for an enzyme. A bond covered by hemicellulose can be digested as soon as the hemicellulose is digested (see Fig 2). An additional consideration for lignin is the fact that lignin polymers are highly branched in nature [48–50]. To model lignin branching in a simple manner, we define a covering fraction that represents the fraction of monomers, for each lignin polymer, that effectively covers the microfibril. The remaining bonds do not take part in the blocking process. In Fig 5, we illustrate the case of 100% covering, which corresponds to linear lignin polymers, and the case of 20% covering, which corresponds to highly branched and complex-shaped lignin polymers. In order to mimic the natural variability of the lignin polymer branching, for each polymer, the covering fraction is randomly selected from a normal distribution of average *μ* and standard deviation *σ*, which are both tunable parameters.

#### Crystallinity

It is well known that cellulose adopts a highly crystalline arrangement [70–72], and that cellulose microfibrils contain crystalline and amorphous regions [73]. While hemicellulose polymers are generally arranged in an amorphous manner [37], hemicellulose crystallinity is still debated within the literature. We therefore choose to not restrict this aspect and consider both amorphous and crystalline regions for hemicellulose too. This is primarily supported by the fact that we currently only consider xylan in our model, and xylan has been shown to partially bind to cellulose microfibrils, thereby adopting a semi-crystalline arrangement [74]. Additionally, there is evidence of hemicellulose adsorption to cellulose interfering with the saccharification process [75]. In the model, we implement every cellulose and hemicellulose polymer as split into difficult to digest “crystalline” regions and more easily digestible “amorphous” regions. The size of the regions may be individually specified for cellulose and hemicellulose, as fractions of the overall cellulose and hemicellulose content respectively. Using the assumption that the order between the neighboring cellulose polymers decreases towards the ends of the microfibril, the crystalline regions are in the center of the microfibril. Additionally, *r*_c,a_ denotes the ratio of the digestibilities for the crystalline regions (*d*_crystalline_) *versus* amorphous regions (*d*_amorphous_):

$$\begin{array}{c} r_{c,a}=\frac{d_{\text{crystalline}}}{d_{\text{amorphous}}}. \end{array}$$ (3)

It is a parameter that may also be tuned for cellulose and hemicellulose individually. It determines, how often a bond in a crystalline region is digested as compared to an amorphous region. Since crystalline regions are more difficult to digest than amorphous ones, *r*_c,a_ lies between 0 (the crystalline region cannot be digested) and 1 (it is equally well digested as the amorphous region).

### 2.4 A quick reminder of the Gillespie algorithm

The saccharification of the substrate is simulated using a Gillespie algorithm. Individual parameters of the system can be varied and measured in an independent and fully controllable manner. The algorithm is a typical method to implement stochastic simulations, and thereby mimic the dynamics of a system by assuming a sequence of randomized events [76]. Here, these events correspond to adsorption of CBH to a polymer end, binding of enzymes to lignin, and reactions of enzymatic digestion. In the Gillespie algorithm we keep track of all and only doable reactions, by only accounting for the bonds accessible to the enzymes. At each step of the sequence of events, both the reaction to take place and its duration are randomly selected. Still, the algorithm ensures that the chance to select a reaction is on average equal to its likelihood to take place. The more reactions may take place, the more frequently an event happens. As an example, considering an available substrate in excess, more events take place per unit of time if the concentration of enzymes increases. The simulation lasts until the chosen amount of events is attained, or until all of the digestible substrate has been digested.

## 3 Simulation conditions

### 3.1 Default simulation conditions

Unless otherwise specified, the simulations presented here were performed at the following default conditions: i) The microfibril length is 200 bonds. ii) The composition of the microfibril is 61.9% cellulose, 9.5% hemicellulose, and 28.6% lignin. These values correspond to the composition of the medium pre-treatment sample in the study by Bura et al. [11]. iii) Throughout, we use the crystallinity fractions we obtained by fitting the experimental data by Bura et al. [11] for medium pre-treatment severity. These are 19% for cellulose and 23% for hemicellulose, while the digestibility ratios (*r*_c,a_) are 0.03 for crystalline cellulose and 0.043 for crystalline hemicellulose. Table 1 summarizes the parameters in ii) and iii). iv) The stochastic noise between individual simulations decreases with increasing microfibril length. For the default microfibril length of 200 bonds, the relative root mean square error between an average over 10 simulations and an average over 100 simulations is around 1%. This means that by performing 100 simulations instead of 10 we improve our results by about 1% only. Therefore, although we choose 100 simulations throughout the paper, in those cases where large amounts of distinct simulation sets are required (Fig 7C and 7D: 10 simulations per pixel), we deem 10 simulations per set to be sufficient. v) The individual enzyme abundances in the system were set to values corresponding to an overall concentration of 5 μM. This choice is motivated and explained in the following paragraph.

**Table 1:** Parameters for the substrate composition, crystallinity fractions, and digestibility ratios (*r*_c,a_) at three pre-treatment severities (low, medium and high). The composition is closely derived from experimental data: the percentages of hemicellulose and lignin are from Bura et al. [11], while the glucose percentages are adapted such that the composition percentages sum up to 1. The crystallinity fractions and digestibility ratios *r*_c,a_ are determined by fitting the experimental saccharification time courses by Bura et al. [11] (see section 4.4).

Pre-treatment severity	Composition (%)			Crystallinity fractions (%)		*r*_c,a_ (%)	
	Cellu	Hemi	Lignin	Cellu	Hemi	Cellu	Hemi
Low	57.0	18.8	24.2	52	68	3.0	4.3
Medium	61.9	9.5	28.6	19	23	3.0	4.3
High	62.7	5.3	31.1	3.1	6.2	3.0	4.3

### 3.2 Enzyme concentration

To specify the number of enzymes in the system we consider both the geometrical properties of our *in silico* substrate and the experimental setup we aim to reproduce in subsection 4.4. The setup is that of the saccharification time courses generated by Bura et al. [11].

#### 3.2.1 Simulation volume

To translate the experimental conditions to our model, we assume that the macroscopic substrate is divided into identical sub-units, which each resemble the simulated structure. Thus, we can estimate the volume surrounding a single sub-unit (*V*_surrounding_). This is done via

$$\begin{array}{c} V_{\text{surrounding}}=\frac{V_{\text{solution}}}{N_{\text{SU}}}-V_{\text{SU}}\;, \end{array}$$ (4)

where *V*_solution_ is the total volume of the solution, *N*_SU_ is the total number of sub-units, and *V*_SU_ is the volume occupied by a single sub-unit. Within the saccharification experiments done by Bura et al. [11], the solution volume for each sample is *V*_solution_ = 50 ml. To determine *V*_surrounding_ we require *N*_SU_ (Number of sub-units) and *V*_SU_ (Volume of a sub-unit).

#### Number of sub-units

With the assumption that all sub-units are identical, the number of sub-units within the substrate is:

$$\begin{array}{c} N_{\text{SU}}=\frac{m_{\text{substrate}}}{m_{\text{SU}}}\;, \end{array}$$ (5)

where *m*_SU_ is the mass of a single sub-unit and *m*_substrate_ is the mass of the whole substrate. In the study by Bura et al., the substrate was diluted at a so-called weight-to-volume consistency of 8%, which corresponds to a total substrate mass of *m*_substrate_ = 4g.

The mass of a single sub-unit depends on the abundance and molecular masses of its constituents, following

$$\begin{array}{c} m_{\text{SU}}=m_{\text{glc}}\cdot N_{\text{glc}}+m_{\text{xyl}}\cdot N_{\text{xyl}}+m_{\text{lign}}\cdot N_{\text{lign}}\;. \end{array}$$ (6)

The numbers of monomers (*N*_glc_, *N*_xyl_, and *N*_lign_) are calculated from the sub-unit length and the percentages of cellulose, hemicellulose and lignin. We use the percentages found by Bura et al. [11] for medium pre-treatment severity for this estimation (see Table 1). The molecular masses for the three monomers (*m*_glc_, *m*_xyl_, and *m*_lign_) are shown in Table 2. Using this, a microfibril with 200 bonds per cellulose polymer has a mass of *m*_SU_ ≈ 3.4 × 10^−18^ g, and the number of sub-units in 4 g of substrate is *N*_SU_ ≈ 1.2 × 10^15^.

**Table 2:** Molecular masses of the constituents of the lignocellulose sub-units, obtained from literature and rounded to three digits. The value for the representative monolignol was taken as the mean value of the molecular masses of the three main monolignols (coumaryl alcohol, coniferyl alcohol and sinapyl alcohol).

Molecule	Glucose	Xylose	Monolignol
**Molecular mass** ($\frac{\text{kg}}{\text{mol}}$)	0.180	0.150	0.180

#### Volume of a sub-unit

The volume occupied by a single sub-unit is calculated via

$$\begin{array}{c} V_{\text{SU}}=d_{\text{SU}}^{2}\cdot \left(N_{\text{bonds}}+1\right)\cdot d_{\text{glc}}\;, \end{array}$$ (7)

where *d*_glc_ is the distance between bonds, assumed to be equal to the diameter of a glucose molecule, *d*_SU_ is the cross-section of a sub-unit, and *N*_bonds_ is its length, counted in bonds. First, we assume that *d*_glc_≈ 1 nm. Second, according to Ding et al. the cross-section of a cellulose bundle of 36 polymers is roughly 5.3 nm × 3.2 nm [57]. In order to include the outer layers of hemicellulose and lignin, we set *d*_SU_ = 10 nm. Third, the bond number *N*_bonds_ is set to 200. We determine that *V*_SU_ ≈ 2 ⋅ 10^−22^m^3^.

With the values of *N*_SU_ and *V*_SU_, we can finally calculate the volume surrounding a single sub-unit: *V*_surrounding_ ≈ 4.26 ⋅ 10^−20^m^3^. This volume corresponds to a cube with a side length *l*_cube_ of roughly 350 nm. The number of enzymes *N*_enzyme_ in the simulated system which correspond to a given concentration *c*_enzyme_ can now be calculated via

$$\begin{array}{c} N_{\text{enzyme}}=c_{\text{enzyme}}\times V_{\text{surrounding}} \end{array}$$ (8)

#### 3.2.2 Choice of concentration

With a highly resolved molecular dynamics approach, Vermaas et al. modeled a system containing 9 lignocellulose microfibrils surrounded by 54 enzymes, in a volume equal to 95 nm × 62.5 nm × 62.5 nm. Within this volume, which is roughly 115 times smaller than ours, 54 enzymes correspond to a concentration of approximately 240 μM. However, we cannot directly compare our situation with that of Vermaas et al., because they consider enzymes which are very closely associated to the substrate at all times. If we were to simply scale up Vermaas’s concentration to our volume, we would have almost 6200 enzymes in our system. In such a crowded case, the assumption of freely diffusing enzymes that we require for the enzyme concentration to be homogeneous and the diffusion time to be short as compared to the reaction time, is no longer viable. The incompatibility of our model with the concentration of Vermaas et al. is confirmed by the fact that they picture a heterogeneous enzyme concentration, while we consider a well homogenized setup.

In the experimental literature, concentrations are not often provided. Instead, the enzyme loading is usually specified in terms of filter paper units (FPU) or overall enzyme mass. The latter can be translated to concentration by knowing the solution volume and the mass of a single enzyme. Park et al. used cellulase cocktails weighing between 15 mg and 150 mg within a solution volume of 50 mL [77]. Cellulases from *Trichoderma reesei* have individual masses around 51 kD (Endoglucanase EG-1: 48 kD [60]; Exoglucanase-1: 54 kD [61]), which would translate to concentrations between 5 μM and 50 μM in the experiments by Park et al. [77]. Within our simulations, we assume a dilute system and therefore use the lower end of this concentration range, i.e., 5 μM. Within our simulated volume *V*_surrounding_ this corresponds to roughly *N*_enzyme_ = 148 enzymes (see Eq 8).

An additional consideration is the relative abundance of the individual enzymes. According to Sinitsyn et al. [7], an efficient cellulase cocktail contains 12-18% of EG, 36-41% of CBH and 8-14% of BGL. These “percentages” do not add up to 100, so we can only obtain ratios from them. Taking the mean value of these 3 ranges respectively (*p*_*EG*_ = 15%, *p*_*CBH*_ = 38.5%, *p*_*BGL*_ = 11%), we can obtain the relative percentages as:

$$r_{\text{EG}}=\frac{p_{\text{EG}}}{p_{\text{EG}}+p_{\text{CBH}}+p_{\text{BGL}}}\approx 23\%$$

$$r_{\text{CBH}}=\frac{p_{\text{CBH}}}{p_{\text{EG}}+p_{\text{CBH}}+p_{\text{BGL}}}\approx 60\%$$

$$r_{\text{BGL}}=\frac{p_{\text{BGL}}}{p_{\text{EG}}+p_{\text{CBH}}+p_{\text{BGL}}}\approx 17\%$$

Furthermore, Agrawal et al. investigated the optimal ratio between Celluclast cellulase, β-glucosidase and xylanases [58], and arrived at ratios of 20.40, 38.43 and 41.16 respectively. As Celluclast is a proprietary cellulase cocktail whose exact content is unknown, we assume it is mostly made of cellulases, and we combine it to β-glucosidase to determine the ratio between cellulases and xylanases. We find ratios of 58.83 and 41.16 respectively. Using the overall number of enzymes stated above (*N*_enzyme_ = 148) and the results of Sinitsyn et al. for the cellulases [7], the default numbers for EG, CBH, BGL and XYL in our simulations follow as:

$$N_{\text{EG}}=20,N_{\text{CBH}}=52,N_{\text{BGL}}=15,N_{\text{XYL}}=61.$$

## 4 Results

Our aim is to understand the effect of the substrate structure on the action of the enzymes as well as their interaction with non-digestible lignocellulose components. To do so, we start from an investigation of the main features of the model. These are the synergism of the enzymes, the influence of lignin, and the impact of the crystallinity. Afterwards, we reproduce experimental saccharification time courses by Bura et al. [11] for different pre-treatment conditions. We semi-quantitatively determine the impact of the substrate characteristics in shaping the saccharification process. Alongside this, we also interpret how pre-treatments affect the substrate structure.

### 4.1 Enzymatic synergism

**Fig 6:**
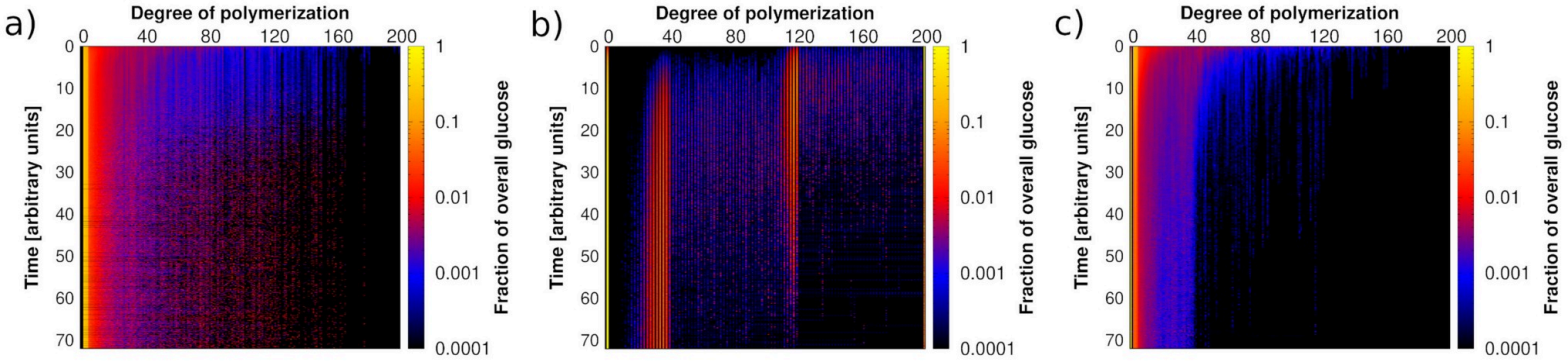
Heatmaps depicting the dynamics over time of the distribution of cellulose polymer degrees of polymerization (DP) for a default microfibril. Time is shown on the ordinate, DP is shown on the abscissa, and color indicates the total amount of glucose that makes up polymers of length DP divided by the total amount of glucose in the system. Present enzymes are xylanase (XYL), together with: EG in (a), CBH in (b), and EG, CBH and BGL all together in (c). In (a), cellotriose accumulates, since in the model EG alone cannot release cellobiose or glucose. In (b) cellobiose progressively accumulates. Finally, in (c), cellobiose can be digested by BGL, and in turn glucose accumulates. Each heatmap represents an average over 100 simulation runs.

The enzyme cocktails used by plant-digesting fungi are effective thanks to the combined action of the individual enzymes [65]. Here we first utilize the model to analyze the action of these enzymes individually, and then collectively, in order to explain and quantify their synergism. The heatmaps shown in Fig 6 depict the distribution of cellulose polymer degrees of polymerization over time, for different enzyme sets. The simulated microfibril is the default case, which includes cellulose, hemicellulose, lignin and crystallinity in the precise amounts specified in subsection 3.1.

When only endoglucanase (EG) and xylanase (XYL) are present (Fig 6A), the distribution of cellulose quickly changes, from initially being made of the longest polymers, to including the shorter ones. This can be explained by the fact that endoglucanase can randomly cut glycosidic bonds along cellulose filaments, which results in polymers of any length. However, since in the model EG does not cut off cellobiose or glucose from the polymers, none of these two are released, and the two leftmost columns of the heatmap remain black throughout the simulation. Instead, cellotriose accumulates over time (third column from left). Symmetrically, no polymers of length 199 or 198 appear (second and third columns from the right).

When only cellobiohydrolase (CBH) and xylanase (XYL) are present (Fig 6B), the distribution moves towards shorter polymers linearly in time, while cellobiose (second column from left) steadily increases. Starting from a monodisperse pool of chain length 200, the digestion by CBH, which can only release cellobiose, leads to even chain lengths. These appear as vertical stripes in the distribution. The linearity of the profile emerges from the processive action of CBH in cutting off cellobiose from any of the two ends of the cellulose polymers. The distribution is also characterized by two patches of increased glucose content around DPs of 40 and 120. This can be explained by the existence of crystalline domains at the center of the microfibril. For medium pre-treatment severity, 19% of the cellulose is crystalline, i.e., 38 bonds within the center of each cellulose polymer. On each side, 81 amorphous bonds remain. A polymer that is digested from both ends until the crystalline domains will have 38 bonds remaining, while a polymer that is only digested from one end will have 119 bonds remaining. These two digestion stages lead to the two observed patches.

Finally, in Fig 6C we simulate the action of a set of cellulases that contain EG, CBH and BGL, together with XYL. We observe that similarly as in Fig 6A under the action of EG, the distribution moves quickly towards shorter polymers. In comparison to Fig 6A, here the cumulative action of CBH contributes to further shifting the full distribution towards short polymers. We also notice that the characteristic stripes arising from the action of CBH in Fig 6B are now absent. Unlike for both Fig 6A and 6B, we hardly see any cellobiose in the system, as this is finally digested into glucose by BGL. The synergism between EG, CBH, BGL and XYL strongly impacts on the dynamics of the distribution of cellulose polymers, and yet, the effect of crystallinity persists, since the patch in the distribution around DP 40 is still visible. This highlights the complexity of the interplay among the enzymes, and between the enzymes and the substrate.

### 4.2 Lignin influence

**Fig 7:**
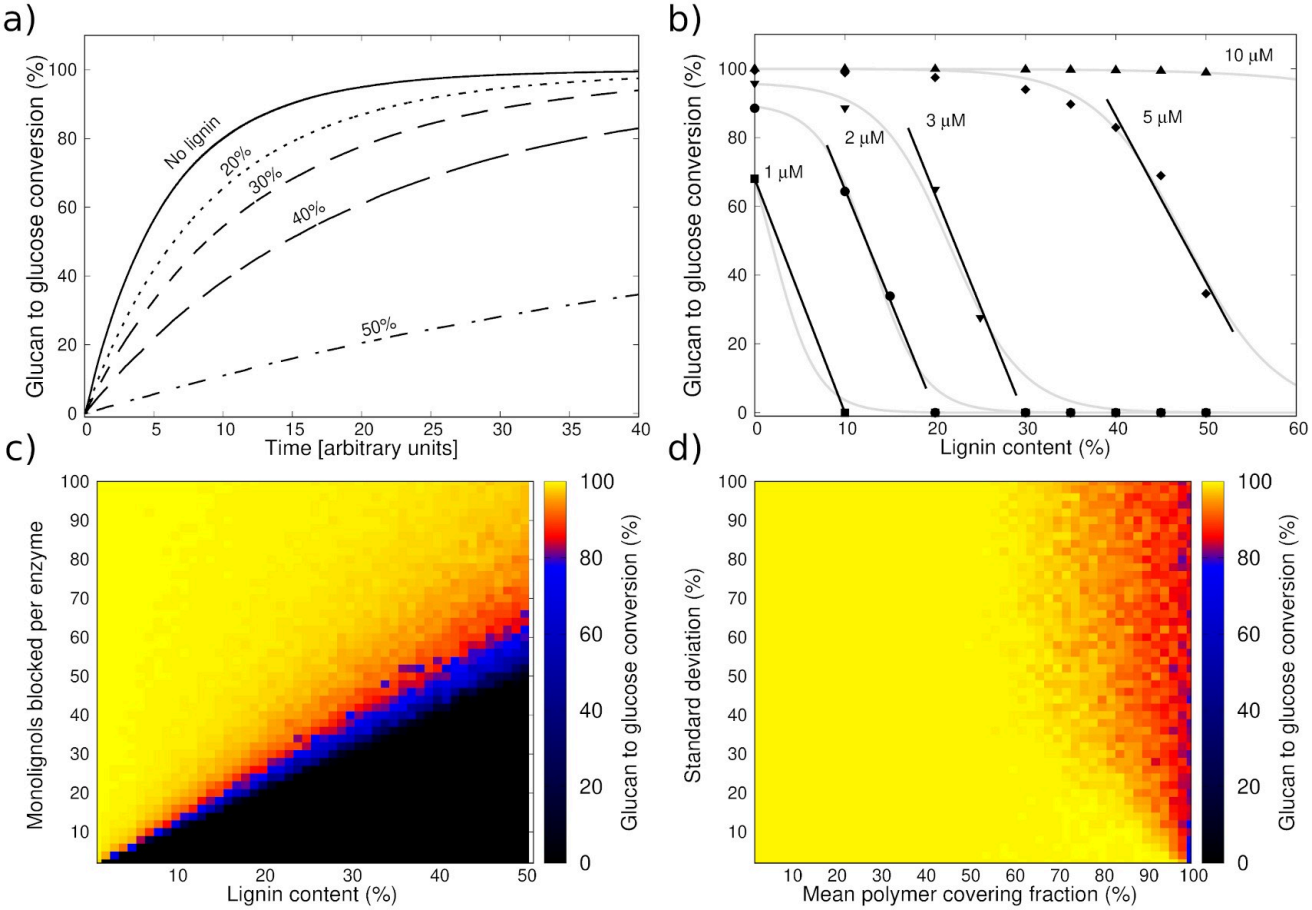
(a) Simulated saccharification time courses for increasing lignin percentage up to a time *t*_end_ = 40 (arbitrary units). Each curve represents an average over 100 simulation runs. (b) Simulated glucan to glucose conversion at time *t*_end_ *versus* lignin content for five different values of the overall enzyme concentration [*E*]. The gray lines trace the inverse logistic behavior, while the black lines indicate the approximately linear intermediate regimes. Each point represents an average over 100 simulation runs. (c) Simulated glucan to glucose conversion at time *t*_end_ *versus* lignin content and number of monolignols involved in the binding of a single enzyme (*N*_lignols,bound_). Enzyme concentration is: [*E*] = 5 μM. (d) Simulated glucan to glucose conversion at time *t*_end_ *versus* mean polymer covering fraction and its standard deviation (see also section 2.3). The lignin content is 50%, and the enzyme concentration is [*E*] = 5 μM. In (c) and (d) each pixel represents an average over 10 simulation runs.

In Fig 7A, we show the saccharification dynamics up to a time *t*_end_ = 40 (arbitrary units), for a substrate of length 200 bonds. It is composed of 50% cellulose, with a varying outer hemicellulose and lignin shell, such that the overall lignin percentage ranges from 0 to 50%. At *t* = *t*_end_, the cellulose within the substrate containing no lignin is almost completely digested. At increasing lignin percentage, the digestion of the substrate takes longer. We observe a steeper decrease in glucan to glucose conversion percentage at *t* = *t*_end_ for the same increase in lignin percentage at higher overall lignin content. This indicates that within our model the dependence of the conversion percentage on the lignin content is non-linear. Upon investigating only the conversion at *t* = *t*_end_ for five different enzyme concentration values (Fig 7B), we see that overall the change in conversion behaves similarly to a logistic decay (gray lines). This is characterized by an initially small slope, followed by an intermediate strong decay towards a conversion of 0%. The behavior within the domain of strong decay can also be approximated as linear, indicated by the trend lines.

By comparing the five curves in Fig 7B, we see that the overall behavior of the glucan to glucose conversion at time *t*_end_ (arbitrary units) depends strongly on the ratio (*r*_E,L_) between the enzyme concentration and the lignin content. This is due to the fact that only a finite number of enzymes can bind to a given amount of lignin. Considering for instance the second-to rightmost curve (diamonds, [E] = 5 μM), at high *r*_E,L_ (leftmost points of the curve) the lignin negligibly influences the action of the enzymes and we observe a plateau. At low *r*_E,L_ (rightmost points of the curve), the lignin strongly disrupts the conversion percentage, and we observe a sharp drop of the curve. By reducing the enzyme concentration, this profile is shifted to the left, such that the strong disruption regime appears at lower lignin content. The trade-off of enzymes that either perform saccharification, or get inactivated by binding to lignin underpins the logistic decay behavior.

Chen and Dixon [78] analyzed the total sugar released by lignocellulose from alfalfa mutants containing different amounts of lignin, both for acid-pretreated samples and untreated samples. They observed an inverse linear relationship between lignin content and glucan to glucose conversion. A similar linear decrease tendency has been observed by Studer et al. [79] for distinct plant materials. They selected different phenotypes of poplar trees based on their lignin content, and also studied their saccharification dynamics. Additionally, Guo et al. investigated the saccharification dynamics of rice straw, bagasse and silver grass [80], which differ in lignin content. They observed negative correlation between the lignin content and initial sugar release rate. Finally, Van Acker et al. [81] studied the saccharification and fermentation properties of field-grown transgenic poplar that were deficient in cinnamoyl-CoA reductase. They concluded that the significant decrease of Klason lignin content agreed with improved saccharification and fermentation yields. With our model, we are able to show a comparable linear decrease, which is highlighted by trend lines in Fig 7B. This supports that in the model lignin is fairly represented by its interactions with enzymes and the blocking of the structure. Importantly, our model offers to investigate the dynamics of these effects throughout the saccharification process and, being a flexible theoretical tool, it allows us to study ranges of lignin content beyond the typical experimental results found in the literature. Screening a larger range of lignin percentages reveals that the experimentally observed linear decrease can possibly be interpreted as the intermediate regime of a logistic decay, which also shows a plateau-like profile at low lignin content.

To further investigate the action of lignin, we separately examine the adhesion effect and the structural blocking effect. Starting with the adhesion effect within Fig 7C, we vary both the lignin content and the number of monolignols which are involved in binding a single enzyme (*N*_lignols,bound_, see also section 2.3). We show the conversion percentage at time *t*_end_ as a heatmap. In the bottom left corner we observe an initially sharp edge between regions of maximal and minimal glucan to glucose conversion, which broadens as the lignin content increases. This highlights the trade-off between the amount of available lignin and its binding capacity: high amounts of lignin that can only bind a small number of enzymes (upper right corner) have a negligible impact on the glucan to glucose conversion, as do low amounts of lignin that can bind a large number of enzymes (lower left corner).

Finally, within Fig 7D, we analyze the structural blocking effect by lignin. We fix the lignin percentage to 50% and vary the mean polymer covering fraction *μ* and its standard deviation *σ* (see also section 2.3). The glucan to glucose conversion percentage at time *t*_end_ is once again shown as a heatmap. For values of *μ* greater than around 50% the impact of lignin in blocking access to the structure is visible. It starts from reducing the glucan to glucose conversion percentage by about 10%, and it takes it down to approximately 50% when lignin is linear (*μ* = 100%). If the lignin content would go beyond 50%, the impact of lignin in blocking access to the structure would increase further.

The impact of lignin on saccharification can be drastic. It depends on several factors that include its abundance, adhesive strength and structure, in particular the covering of the cellulose microfibril by the lignin polymers. Moreover, the interpretation of the impact of lignin must take the enzyme abundance into consideration. Our results suggest that the well-accepted linear decrease pattern of the final glucan to glucose conversion *versus* lignin content is in fact the intermediate regime of a more complex profile. The values of lignin content at which this regime emerges highly depend on the enzyme concentration.

### 4.3 Substrate crystallinity

**Fig 8:**
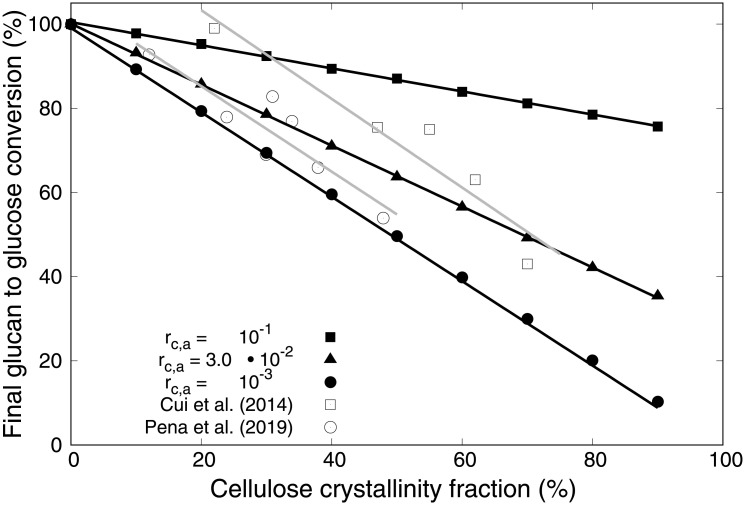
Simulated final glucan to glucose conversion *versus* crystallinity fraction for different ratios (*r*_c,a_) between the digestibilities of the crystalline and the amorphous regions. For each value of *r*_c,a_ we observe a linear decrease in final glucan to glucose conversion percentage for increasing crystallinity fraction, whose slope becomes steeper as *r*_c,a_ decreases. For *r*_c,a_ = 10^−3^ we observe inverse proportionality in addition to linearity. Each simulated point represents an average over 100 simulations. Also shown are experimental data by Cui et al. [71] (empty squares) and Pena et al. [72] (empty circles).

The impact of cellulose crystallinity on the saccharification dynamics is a matter of recent focus [71, 72]. To investigate the influence of the crystallinity, in Fig 8 we consider three sets of simulations for a microfibril of default composition (see Table 1, medium pre-treatment severity). The difference between them lies in the ratio (*r*_c,a_) between the digestibilities of the amorphous and crystalline regions. For each set, the fraction of crystalline regions is varied between 0 and 90% of the respective substrate, and the resulting final glucan to glucose conversion percentages are compared. The latter are measured at time *t*_end_ = 72 (arbitrary units) for later comparison with experimental data (see section 4.4). We observe a linear decrease of the final glucan to glucose conversion with the increase in crystallinity fraction. The corresponding slopes become steeper at lower *r*_c,a_. We observe inverse proportionality in addition to linearity when *r*_c,a_ = 10^−3^, i.e., when the crystalline regions are almost impossible to digest compared to the amorphous ones.

Also shown in Fig 8 are experimental data by Cui et al. [71] and Pena et al. [72]. Cui et al. investigated the influence of four different pre-treatment methods (ionic liquid, ethylenediamine, glycerol, and sodium hydroxide) on the crystallinity of α-cellulose samples, and further investigated their glucan to glucose conversion depending on the crystallinity. They observed an inverse linear relationship between the measured crystallinity index and the conversion. Pena et al. on the other hand focused exclusively on the ionic liquid pre-treatment method, and investigated the cellulose crystallinity after different pre-treatment durations. Their measurement of the glucan to glucose conversion also shows an inverse linear relationship. Importantly, both sets of data are reasonably well matched by the model, even though the experimental setup varies considerably between them. We remark that the curve by Cui et al. is shifted to the right, with complete glucose conversion achievable despite about 22% of crystalline cellulose. This might suggest a slightly different definition of the crystallinity index.

### 4.4 Comparison of the model to experimental time course data for pre-treated plant cell wall material

#### Fitting procedure

In the model, individual parameters of the system can be varied and measured in an independent and fully controllable manner. In order to investigate the capability of the model to precisely reproduce experimental saccharification time courses, we devised a parameter fitting algorithm. It can be applied to any parameter of the model to optimize it. Among the set of all the parameters which characterize the model, we choose to optimize only a subset of parameters *p*_i_. The algorithm works in recursive generations, that each build on the preceding one. Each generation contains a number of distinct subsets, whose parameters vary within a percentage Δ from the preceding generation of parameters (*p*_i,old_). More precisely, each subset’s parameters *p*_i_ are randomized, but lie within the respective interval [*p*_i,old_ − Δ ⋅ *p*_i,old_; *p*_i,old_ + Δ ⋅ *p*_i,old_]. The total number of subsets within a single generation can be freely specified at the start of the algorithm.

At each generation, the saccharification process is simulated for all the subsets of parameters, and the glucan to glucose and the xylan to xylose conversions are recorded. For each subset, we determine the average curves by running several independent simulations, whose number is a parameter that can be freely specified at the start of the fitting algorithm. Then, the difference between the average simulated and the experimental results is measured for successive time points along the saccharification curves. This is done for glucose and xylose curves separately, and can be applied to multiple experimental datasets simultaneously, for instance different pre-treatments. The resulting errors are then combined within an average error between theory and experiments, which depends on all different glucose and xylose curves.

After the error between simulated and experimental curves has been measured for each subset of parameters, the best fitting candidate, with the lowest error, is found. If this error is higher than that of the subset of parameters *p*_i,old_, the old parameters are kept and used again as a starting point for the subsequent generation. Otherwise, the parameters of the new subset are retained. Additionally, the gradient within the parameter space between the old and the new parameters is calculated. This direction is followed for the next generation. If the error has not been reduced after this next generation, the algorithm reverts to randomly assigning parameter values depending on Δ. After multiple generations, this hybrid procedure that mixes random and directed search leads to a local or global optimum in precision.

#### Experimental approach

**Fig 9:**
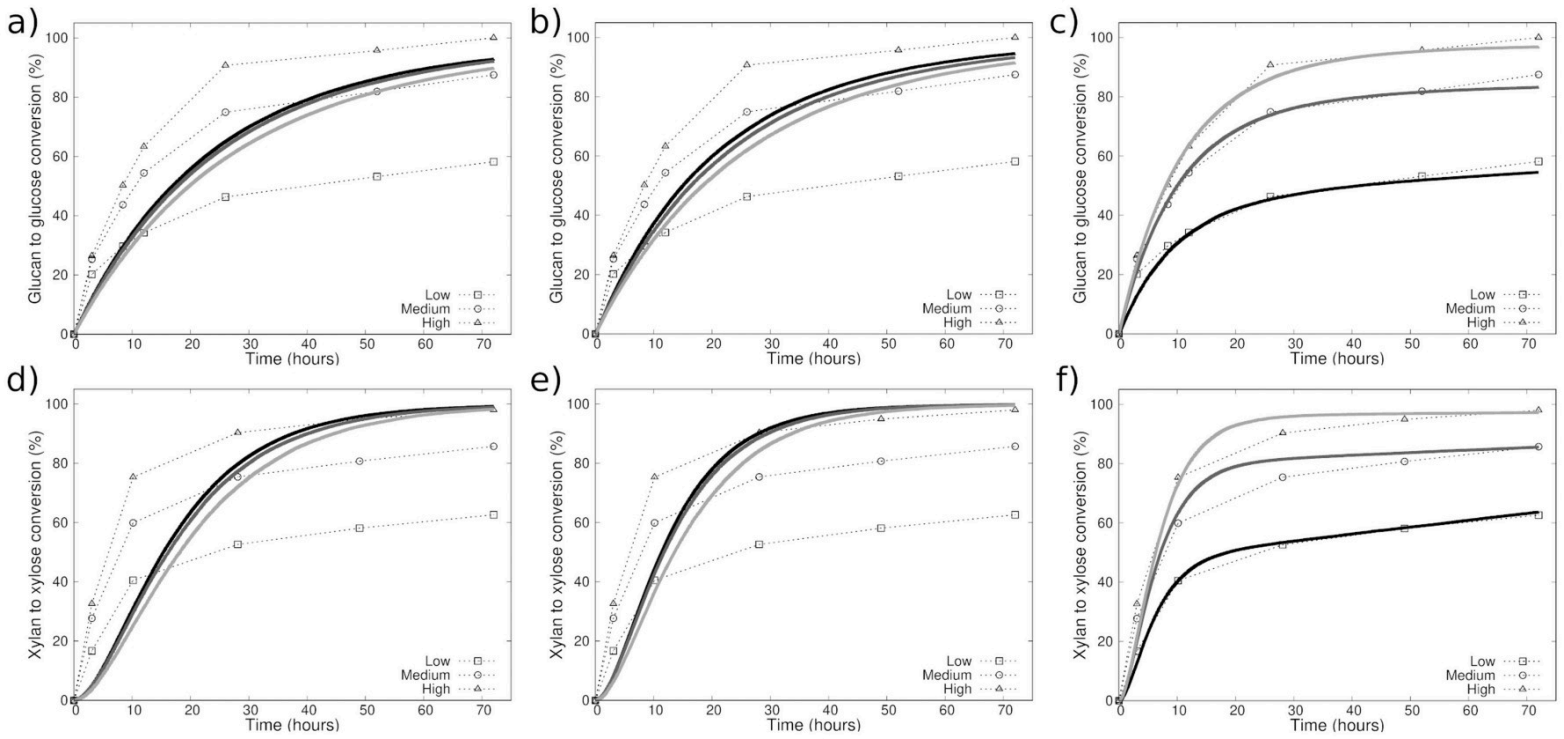
Experimental data (dotted lines) and best simulation fits of the saccharification time courses for three different pre-treatment severities (low: Black lines, medium: Dark grey lines, high: Light grey lines). (a) and (d) The substrate has no structure and all polymers are freely floating within the medium. (b) and (e) The cellulose polymers form a microfibril, which is surrounded by hemicellulose and lignin. However, the crystallinity of the substrate is discarded. (c) and (f) The substrate crystallinity is additionaly included, which substantially improves the fits. Each curve represents an average over 100 simulation runs. The experimental data shown here are from Bura et al. [11].

To demonstrate the ability of our model to reproduce experimental saccharification time courses, we use results by Bura et al. [11]. They studied the influence of a steam pre-treatment process on the saccharification dynamics of plant material from corn stover. For this, they first subjected their samples to three different pre-treatment severities, denoted “low”, “medium” and “high”. The severities were characterized both by the temperature during the pre-treatment and the overall duration of the pre-treatment. Following the pre-treatment, the samples were examined with respect to their composition of cellulose, hemicellulose and lignin. Table 1 outlines the substrate compositions resulting from the three pre-treatment severities. Then, a cocktail of cellulases and xylanases was added to the samples, and the glucan to glucose and xylan to xylose conversion percentages were periodically measured over 72 hours (see dotted lines in Fig 9). Higher conversion percentages were observed for higher pre-treatment severities, and it was concluded that the concomitant reduction of the xylan content was the main influence in determining the saccharification dynamics.

#### Simulation results

The findings of Bura et al. suggest that the substrate composition in terms of cellulose, hemicellulose and lignin determines the saccharification dynamics. We investigate this idea within our model by using the composition data provided by them and attempting to optimize the parameters of the model to reproduce the experimental saccharification curves. Our goal is to find a set of parameters with which all six experimental curves (glucose conversion and xylose conversion for each of the three pre-treatment severities) can be reproduced by only adapting the substrate composition to the respective pre-treatment conditions (see Table 1). Starting from the hypothesis that the composition of the substrate solely influences the dynamics, we progressively additionally consider the effect of its structural properties. Thus, in the following we consider three situations, and for each we show the simulated saccharification time courses with the experimental data in Fig 9. In Fig 9A and 9D, and Fig 9B and 9E respectively, we use the fitting algorithm to optimize the enzyme rates of reaction and the rate of adhesion to lignin. In the Fig 9C and 9F, we additionally optimize the crystallinity fractions and the ratio (*r*_c,a_) between the digestibilities of crystalline and amorphous regions. In each of these three independent cases, all optimized parameters are optimized simultaneously.

In Fig 9A and 9D, we simulate a substrate in which all polymers are assumed to freely float in the medium instead of being arranged within a microfibril. Therefore, every digestible bond (both cellulose and hemicellulose) is accessible from the beginning of the simulation. As can be seen, it is not possible for us to find a set of parameters capable of accurately reproducing the data for this situation. In Fig 9B and 9E, we consider a spatially structured microfibril made of 36 cellulose chains and surrounded by hemicellulose and lignin like in Fig 1. However, crystallinity properties are so far discarded. Such hypotheses do not yield satisfying results either. The best fitting glucose conversion curves (solid lines in Fig 9B) are almost identical for all three pre-treatment severities, and are closest to the data for medium pre-treatment. Similarly, none of the three lines lies close to the respective experimental data for the xylose conversion curves (solid lines in Fig 9E). Therefore, we conclude that the substrate composition alone does not enable reproducing and explaining the experimental data, and neither does considering the substrate structure without distinguishing between crystalline and amorphous regions.

To elucidate this problem, we incorporate more advanced structural properties of the substrate, and so the crystallinity is included in the third case. The crystallinity fractions and the ratios (*r*_c,a_) between the digestibilities of crystalline and amorphous regions are optimized, and are reported in Table 1. As can be seen in Fig 9C and 9F, the agreement between simulations and experiments indeed improves substantially. The simulated glucan to glucose conversion curves (solid lines in Fig 9C) fit the experimental data well, as do those of xylan to xylose (solid lines in Fig 9F). The crystallinity fractions found decrease drastically with increasing pre-treatment severities. This makes sense if one considers the different temperatures used within the three pre-treatment severities. The order of crystalline structures is generally reduced for increasing temperature, and therefore a higher severity leading to a reduction in crystallinity fraction is plausible. In addition, as discussed in section 4.3, it has already been demonstrated experimentally that the cellulose crystallinity influences the saccharification dynamics [71, 72]. Noticeably, here the inclusion of both cellulose and hemicellulose crystallinity is necessary for the fits of the experimental data to be optimal.

Within the simulation scheme, our results imply a significant influence of the crystallinity on the saccharification dynamics and final conversion percentage. The crystallinity allows to both reproduce accurately the experimental data and to explain the differences between the dynamics for the different pre-treatment severities. Importantly, our results also support the hypothesis by Simmons et al. [74] that xylan adopts a semi-crystalline shape around cellulose microfibrils.

## 5 Discussion and conclusions

The enzymatic digestion and fermentation of otherwise unused lignocellulosic biomass is an attractive alternative towards facing the worldwide challenges of energy supply and resource shortage [1]. However, further research is required before the process can be economically viable in industrial use. In particular, the impact of the structure properties of the substrate on the saccharification recalcitrance requires further investigations. While saccharification is intensively investigated from an experimental angle [10–12, 34, 72, 82], computational models that focus on investigating the substrate structure while simulating the whole enzymatic digestion process are scarce, and insufficiently compared to experimental data [26–29].

In this study we have built and analyzed a computational model which simulates the dynamics of the enzymatic saccharification of a cellulose microfibril surrounded by hemicellulose and lignin, which is spatially resolved at the scale of substrate monomers. The model considers both the abundance and arrangement of the polymers, and effects such as enzyme adhesion to lignin, lignin induced structural blocking and the crystallinity of the substrate. It relies on a stochastic Gillespie algorithm to simulate the saccharification dynamics of the system over time. Furthermore, it offers to freely tune the enzyme cocktail composition in terms of cellulases and xylanases, and to investigate both their individual action and their synergism. Even though the model is a coarse-grained and simplified representation of the biological system, it nonetheless retains essential features. It semi-quantitatively reproduces experimental data, and even suggests new explanations for their interpretation. Thereby, we demonstrate the strength of the model and its reuse potential for theory enriched experimental analyses.

Within the model, we keep track of all polymers and thus visualize the action of the enzyme cocktail in great detail. In particular, we can underline the current understanding of the synergism exhibited by them. Cellobiohydrolase (CBH) can only digest the ends of cellulose polymers, and therefore requires endoglucanase (EG) to generate more of these ends by cutting the initially long polymers into a large number of shorter ones. On the other hand, CBH is required to generate cellobiose. Finally, β-glucosidase (BGL) is the enzyme leading to the release of glucose. We have simulated the action and dynamics of the cellulases in detail, but have treated the action of xylanases in a coarse-grained fashion by only including a single representative xylanase enzyme (XYL). A possible expansion of the model would be to consider the diversity and complexity of both the xylanases and the hemicellulose, for instance by including different types of sugars and the corresponding xylanase sub-types.

We have implemented known effects of lignin into our model, i.e., its adhesive properties towards enzymes and its structural blocking function. The analysis of the influence of these effects shows an inverse linear dependence between lignin content and final glucan to glucose conversion percentage, in agreement with experimental observations [78–81]. The model additionally suggests that the dependence may only be linear within a finite range of lignin content that is the intermediate regime of a more complex logistic decay. Upon selectively varying the strength of the effects associated to lignin (adhesion and structural blocking), we show that the simulated glucan to glucose conversion percentage strongly depends on the relative amount of enzymes and lignin in the system. It is also impacted by the number of monolignols involved in binding a single enzyme, which directly reflects the finite binding capacity of lignin. In addition to investigating the final glucan to glucose conversion percentage dependence on the overall lignin content, Studer et al. sorted their lignocellulosic materials into two groups [79]: those whose lignin S/G ratio (S: syringyl; G: guaiacyl) was below 2.0, and those whose S/G ratio was above 2.0. They observed a steeper linear dependence of the glucose release on the lignin content for lower S/G ratios. This suggests that guaiacyl units have a stronger influence on the recalcitrance of the material, possibly because they have a higher affinity for binding enzymes than syringyl units do. As another future direction, we could investigate and possibly quantify this by diversifying the representative monolignol, which is currently the placeholder for lignin monomers in our model. The latter could be replaced with defined S- and G- units, which would exhibit differing adhesion strength.

We choose to define the substrate crystallinity as the inverse of the digestibility by splitting the substrate into easily digestible “amorphous” and difficult to digest “crystalline” regions. Our results show an inverse linear dependence between the cellulose crystallinity and the final glucan to glucose conversion percentage, which is in good agreement with experiments done by Cui et al. [71] and Pena et al. [72]. A further point of interest is that Cui et al. have proposed that the digestibility of crystalline cellulose does not only depend on the crystallinity index measured via X-ray diffraction (the analogue to the crystallinity fraction within our model) [71]. They suggest an additional contribution by the crystal allomorph of the cellulose substrate, i.e., the shape of the microfibril. This effect of the allomorph type on the saccharification dynamics could be investigated within our model by changing the shape of the simulated microfibril.

We also investigated the impact of the structural properties of the substrate by attempting to reproduce experimental time courses by Bura et al. [11] in three different situations: a substrate without structure, a substrate with structure but without crystallinity, and a substrate with both structure and crystallinity. We were unable to find suitable parameters to reproduce the experimental data for the first two situations, but obtained good agreement for the third one. This indicates that, while the substrate composition and the arrangement of the polymers are important, they are not sufficient to interpret the saccharification dynamics, which additionally requires to consider the crystallinity of the substrate. Our results also show that the crystallinity is reduced with increasing pre-treatment severity. Furthermore, the inclusion of crystallinity for both cellulose and hemicellulose was necessary to obtain optimal agreement with the experimental data. This finding supports the semi-crystalline arrangement of xylan around cellulose microfibrils, which was proposed by Simmons et al. [74].

The model captures some of the essential properties of lignocellulose saccharification that are known or hypothesized in literature. We are confident that its flexibility makes it a general platform that allows vast possibility of further development. For instance, the model could permit us to investigate different plant mutants, tissues and enzyme abundances, and kinetics.
